# Statistical and ANN Modeling of Corrosion Behavior of Austenitic Stainless Steels in Aqueous Environments

**DOI:** 10.3390/ma18184390

**Published:** 2025-09-19

**Authors:** Kwang-Hu Jung, Seong-Jong Kim

**Affiliations:** 1 Division of Cadet Training, Mokpo National Maritime University, Mokpo 58628, Republic of Korea; khjung@mmu.ac.kr; 2 Division of Marine Engineering, Mokpo National Maritime University, Mokpo 58628, Republic of Korea

**Keywords:** corrosion prediction, austenitic stainless steel, linear regression, artificial neural network, pitting potential

## Abstract

This study applies statistical approaches utilizing linear regression and artificial neural networks (ANNs) to predict the corrosion behavior of austenitic stainless steels (316L, 904L, and AL-6XN) under various environmental conditions. The environmental variables considered include temperature (30–90 °C), chloride ion concentration (20–40 g/L), and pH (2–6). Analysis of variance (ANOVA) confirmed that the input variables, including the Pitting Resistance Equivalent Number (PREN ranging from 24 to 45), significantly affect the critical pitting potential. The influence of the variables was ranked in the order: PREN, temperature, pH, and chloride ion concentration. A linear regression model was developed using significant factors and interactions identified at the 95% confidence level, achieving a predictive performance with R^2^ = 0.789 for critical pitting potential. To predict potentiodynamic polarization curves, an ANN based on supervised learning with backpropagation was employed. The ANN model demonstrated a remarkably high predictive performance with R^2^ = 0.972 in complex corrosion environments. The predicted polarization curves reliably estimated electrochemical characteristics such as corrosion current, corrosion potential, and pitting potential. These results provide a valuable tool for predicting and understanding the corrosion behavior of stainless steels, which can aid in corrosion prevention strategies and material selection decisions.

## 1. Introduction

Austenitic stainless steels are widely used in various industries, including marine, offshore, chemical processing, and power generation, due to their excellent mechanical properties and corrosion resistance. However, under aggressive environment like seawater, these materials are susceptible to localized forms of corrosion such as pitting [1] and crevice corrosion [2]. Pitting corrosion can lead to catastrophic failures because it is difficult to detect and can propagate rapidly once initiated. Therefore, understanding and predicting the corrosion behavior of austenitic stainless steels is critical to ensuring the integrity and service life of structures and components made from these materials.

Traditional methods for assessing corrosion resistance involve experimental testing, such as potentiodynamic polarization measurements, which can be time-consuming and costly. These methods also require extensive laboratory work to evaluate the effects of various environmental factors like temperature [3,4], chloride ion concentration [5,6], and pH [7]. While empirical models have been developed to estimate corrosion rates and surface corrosion potential, they often lack the ability to accurately predict corrosion behavior under complex environmental conditions due to the nonlinear and multifactorial nature of the corrosion process.

Recent advancements in computational techniques have opened new avenues for predicting material behavior [8,9]. Statistical methods such as linear regression have been employed to establish relationships between input variables and corrosion responses. However, linear models may not capture the complex interactions and nonlinearities inherent in corrosion phenomena. Artificial neural networks (ANNs), with their ability to model complex nonlinear relationships, offer a promising alternative. ANNs have been successfully applied in various fields of materials science and engineering to predict properties and behaviors based on input parameters [10,11,12,13,14].

Several studies have explored the use of ANNs for corrosion prediction. Ji et al. [15] developed an ANN model to predict corrosion rates of steel in dynamic environment, demonstrating improved accuracy over traditional empirical models. Akhlaghi et al. [16] utilized ANNs to estimate the pitting corrosion of steels, considering factors such as soil characteristics and pipe type of buried transmission pipelines.

The Pitting Resistance Equivalent Number (PREN) is a widely accepted parameter used to estimate the pitting corrosion resistance of stainless steels based on their chemical composition, particularly the contents of chromium, molybdenum, and nitrogen. However, PREN alone does not account for environmental factors that significantly influence corrosion behavior. Incorporating environmental variables into predictive models is essential for a more accurate assessment of corrosion risk.

In this study, statistical models were developed using linear regression and artificial neural networks to predict the pitting potential and polarization curves of austenitic stainless steels (316L, 904L, and AL-6XN) under various environmental conditions. By considering factors such as temperature, chloride ion concentration, pH, and PREN, we seek to establish reliable predictive tools that can aid in material selection and corrosion prevention strategies. The linear regression model will help identify the significant factors and their linear relationships with the pitting potential, while the ANN model will capture the nonlinear behaviors and interactions among the variables to predict the entire polarization curve.

This study involves conducting potentiodynamic polarization tests to generate experimental data across a full factorial design of experiments, ensuring that all combinations of factors and levels are considered. Statistical analysis, including analysis of variance (ANOVA), is performed to evaluate the significance of each factor and interaction. The models are then validated using separate datasets to assess their predictive performance.

By integrating statistical methods and machine learning techniques, this study contributes to the advancement of predictive corrosion modeling and provides valuable insights into the corrosion mechanisms of austenitic stainless steels under diverse conditions.

## 2. Materials and Methods

### 2.1. Materials and Specimen Preparation

In this study, the stainless steels used were STS 316L, 904L, and AL-6XN, with their chemical compositions provided in Table 1. For stainless steels, the Pitting Resistance Equivalent Number (PREN), which indicates resistance to localized corrosion based on chemical composition, can be calculated using Equation (1) [14]. To quantify the corrosion characteristics of each stainless steel as variables, the PRENs were applied; the PRENs of 316L, 904L, and AL-6XN are 24, 34, and 45, respectively. Each specimen was machined to dimensions of 20 mm × 20 mm, polished up to #600 grit SiC abrasive paper, cleaned with distilled water, and stored in an oven.

(1) PREN=%Cr+3.3%Mo+16%N

### 2.2. Potentiodynamic Polarization Test

Potentiodynamic polarization tests were conducted to evaluate the electrochemical properties of each specimen under various experimental variables. A three-electrode corrosion cell was configured using a working electrode (specimen with an exposed area of 1.36 cm^2^), a reference electrode (Ag/AgCl sat. KCl), and a counter electrode (platinum mesh of 20 mm × 20 mm size). Polarization was performed at a 1.67 mV/s scan rate, starting from −0.25 V vs. the open-circuit potential (OCP) up to a maximum of 1.2 V, after an initial stabilization period of 1800 s.

### 2.3. Statistical Approach

To obtain the training, validation, and test data for the ANN model, a full factorial model with four factors at three levels was designed. It includes experiments for all combinations of factors and levels [17]. This approach allows for the detection of main effects and interactions, enabling the derivation of regression equations with high reliability for various combinations of levels [18]. The full factorial design used in this study is presented in Table 2 and Table 3. To adjust the levels of each factor, the Cl^−^ ion concentration was controlled using NaCl, and the pH was adjusted using hydrochloric acid. Each factor level was normalized to a value between 0 and 1 using Equation (2).

(2) $$X=(x-x_{min})/(x_{max}-x_{min})$$

Here, *x* is the normalized value and $x_{max}$ and $x_{min}$ are the maximum and minimum values of each level. In the full factorial design with four factors at three levels, a total of 81 (3^4^) experiments were required. To ensure the reliability of each test result, experiments were performed in duplicate. The influence and significance of each factor and level were evaluated through analysis of variance (ANOVA) at a 95% confidence level [19]. Statistical analysis of the full factorial design and data was conducted using Minitab^®^ 19 software (Minitap, LLC, State College, PA, USA).

### 2.4. ANN

**Figure 1:**
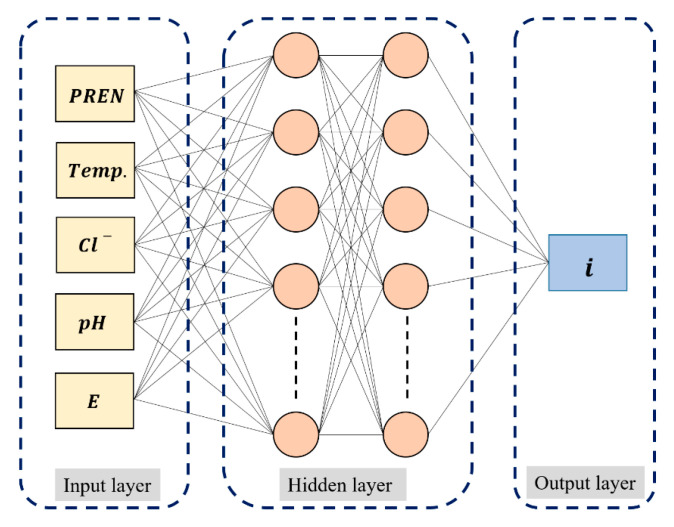
Schematic diagram for ANN architecture used for modeling polarization curves.

ANN method employing the backpropagation algorithm for supervised learning was utilized. Figure 1 illustrates the general structure of the neural network, which is a multilayer perceptron (MLP) consisting of an input layer, hidden layers, and an output layer. The activation functions used for each hidden layer were the sigmoid and hyperbolic tangent (tanh) functions. The data were divided into training, validation, and test sets at ratios of 80%, 10%, and 10%, respectively, and were randomly partitioned in each training process. The design and implementation of the neural network were carried out using Python 3.7 and the TensorFlow 2.0 library. The cost function for training process was the mean squared error (MSE) as shown in Equation (3). Here, n is the number of outputs in the training set, X(t) is the actual value of the data, and $X^{\prime }(t)$ is the predicted value from the model. For the optimization of the cost function, the Adam (Adaptive Moment Estimation) algorithm, known for its superior performance, was employed.

(3) $$\frac{1}{n}\sum_{1}^{n}{\left(X\left(t\right)-X^{\prime }\left(t\right)\right)}^{2}$$

## 3. Results and Discussion

### 3.1. Potentiodynamic Polarization Curves

**Figure 2:**
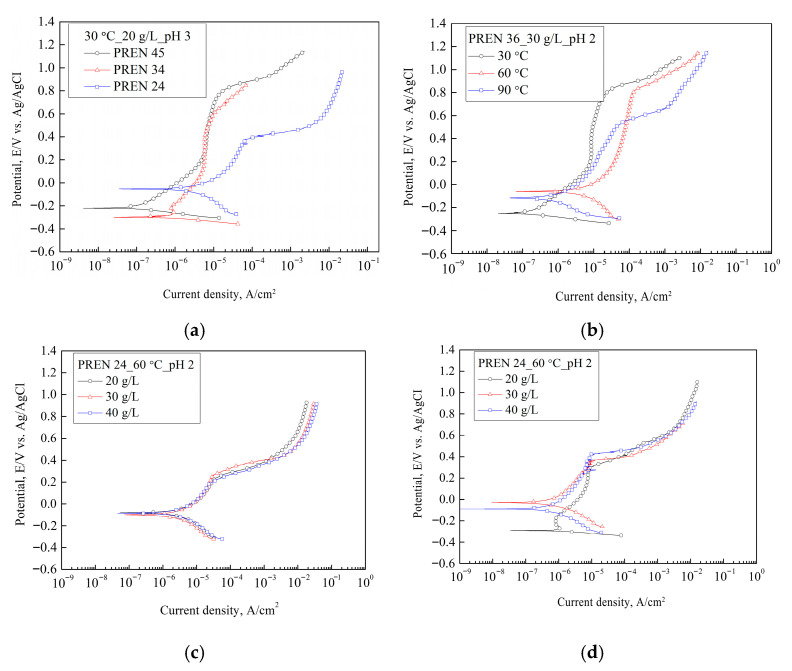
Potentiodynamic polarization curves with variable (**a**) PREN, (**b**) temperature, (**c**) Cl^−^ concentration, (**d**) pH.

Figure 2 presents the polarization curves corresponding to specific factor levels to observe the impact of factor levels on changes in pitting behavior. In each graph, the levels of other factors are held constant except for the specific factor being varied. The critical pitting potentials obtained from each polarization curve, along with the current densities at each potential, were used as training data for linear regression and ANN. Generally, the polarization curves were divided into an anodic region where current density increases with rising potential, a plateau region of constant current density, and a region of rapid current density increase above a certain potential [20]. This behavior allows for the distinction of activation, passive, and passive breakdown regions in the anodic polarization as the potential increases [14,20]. The pitting corrosion can be predicted by comparing the pitting potential with the corrosion potential. Alloys with more noble pitting potentials typically exhibit higher pitting resistance [14,20]. On the other hand, the pitting potential is located in a less noble direction than the corrosion potential or is similar to it, the material is more susceptible to pitting corrosion. In the polarization test results corresponding to the full factorial design presented in Table 3, all pitting potentials were located in a more noble direction than the corrosion potentials, showing varying pitting potentials depending on the factor levels. As shown in Figure 2a,b, the polarization curves exhibited clear differences according to the levels of the pitting resistance equivalent number (PREN) and temperature. In contrast, Cl^−^ concentration and pH did not showed significant differences with changes, as depicted in Figure 2c,d. This indicates that while each factor and its levels influence the polarization characteristics, the degree of their impact varies.

### 3.2. Significance of Input Variables

In a four-factor model, the maximum interaction can extend up to the fourth order (term). As the order is more increases, the fit of the model to the data improves. However, the complexity of interpreting the model also increases significantly, and overfitting to the data may occur [17,21]. Therefore, the model should include appropriate orders and interactions based on the coefficients of determination (R^2^) and significance of each term [22,23]. Table 4 shows the coefficients of determination corresponding to the maximum interaction orders in the full factorial model. The linear model without interactions exhibited a R^2^ of 0.789 and an adjusted R^2^ of 0.777. Models with second-order (quadratic) interactions or higher showed very high R^2^ of 0.97 or above. The model with fourth-order (quaternary) interactions had a R^2^ of 0.996, indicating a fit close to 1. Considering the R^2^ and the ease of interpretation, the model was designed to include second-order interactions.

Table 5 presents the analysis of variance (ANOVA) results for significant factors at the 95% confidence level. ANOVA [24,25,26,27] represents the dispersion of characteristic values as the sum of squares (SS) and decomposes this total sum into sums attributable to experimental factors, identifying those factors that have a particularly large effect compared to the error term. The total sum of squares can be divided into the sum of squares due to deviations of each process variable and the sum of squares due to error. The percentage contribution of each process variable’s sum of squares to the total sum of squares indicates the contribution of that variable to the overall dispersion of characteristic values. Additionally, the F-value, defined as the ratio of the mean square (MS) of a process variable to the mean square of the error, indicates the importance of that process variable relative to the error.

Here, the mean square is defined as the sum of squares of each factor divided by its degrees of freedom (DF). The most critical value in ANOVA is the *p*-value, which determines the significance of factors at the 95% confidence level, using 0.05 as the criterion. If the *p*-value is 0.05 or less, the factor is considered significant at the 95% confidence level, meaning that the levels of that factor have a significant effect on changes in the response variable. In the case of blocks, the *p*-value was 0.926, indicating that the block effect was not significant. This implies that repeated experimental values at the same level do not affect the model interpretation. Based on the ANOVA results, factors that satisfy the confidence level and their influences were identified in the full factorial design.

**Figure 3:**
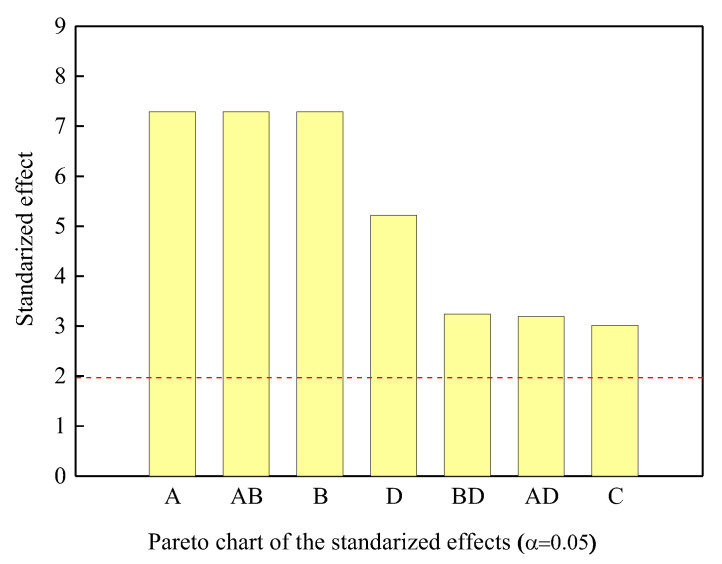
Pareto chart of the standardized effects of significant factors and interaction on critical pitting potential.

**Figure 4:**
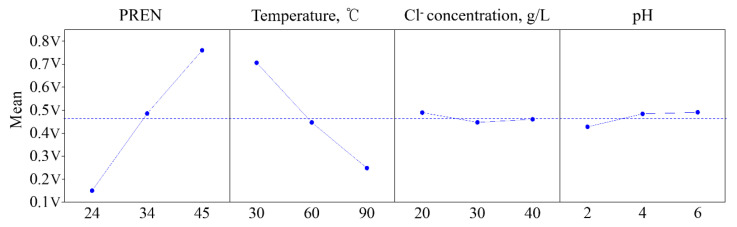
The main effect plots for the mean of critical pitting potential values.

Figure 3 and Figure 4 present Pareto charts illustrating the influence and significance of each input factor. The Pareto chart represents the contribution of each factor to the total contribution rate and can be calculated using Equation (4) [24]. In ANOVA at the 95% confidence level (red dashed line in Figure 3), factors A, B, D, and interactions A·B, B·D, A·D were found to be significant. The order of influence was identified as A (PREN), B (Temperature), C (pH), and D (Cl^−^ ion concentration). This result is consistent with the polarization curves shown in Figure 2. The Cl^−^ concentration increased from the minimum level (20 g/L) to the maximum level (40 g/L), the pitting potential showed an average decrease of about 30 mV. This suggests that the critical Cl^−^ concentration causing a sharp decrease in pitting potential is considered to be below 20 g/L. According to the literature by other researchers, Malik et al. [25] reported a decrease of 200–300 mV in the pitting potential of 316L stainless steel as the chloride ion concentration increased from 100 to 5000 ppm. Leckie and Uhlig [26] observed a decrease of 100 mV in the pitting potential of 18-8 stainless steel over a range of 0.1–1.0% chloride concentration. Ramana et al. [27] reported a decrease of about 40–60 mV in pitting potential with changes in chloride ion concentration from 17,500 to 70,000 ppm at various levels of temperature (20–60 °C) and pH (1.23–5.0). As the pH decreased from the maximum level (pH 6) to the minimum level (pH 2), the pitting potential showed an average decrease of about 64 mV. In acidic environments, a decrease in pH shifts the pitting potential in the less noble direction. However, it has been confirmed through other studies that the degree of this shift does not show a significant difference [25,27,28]. Thus, the changes in pitting potential with variations in chloride ion concentration and pH were consistent with the results reported in other studies.

(4) $$P_{i}=\left(\frac{a_{i}^{2}}{\sum_{i\neq 0}a_{i}^{2}}\right)\times 100\%$$

### 3.3. Prediction of Pitting Potential Using Mathematical Regression Model

Through ANOVA, a regression equation was derived comprising factors and interactions that significantly influence the pitting potential. The first-order polynomial linear model (multiple-linear regression) consisting of four independent variables (*x*) and a dependent variable (*y*) can be expressed as Equation (5).

(5) $$y_{i}=B_{0}+\Sigma_{j=1}^{k}\cdot x_{ij}i=1,2,\cdots n$$

Here, $\beta_{0}$ and $\beta_{i}$ are the bias and weights of the mathematical model, and $x_{ij}$ are the input variables for each term. Table 6 presents the mathematical regression model capable of producing continuous results, along with its coefficient of determination, excluding terms for specific factor levels. The coefficient of determination for the regression model did not show significant differences depending on the inclusion of second-order or higher interactions. Instead, the linear regression model exhibited a higher coefficient of determination, indicating that a linear relationship between factor levels and pitting potential is more appropriate.

**Figure 5:**
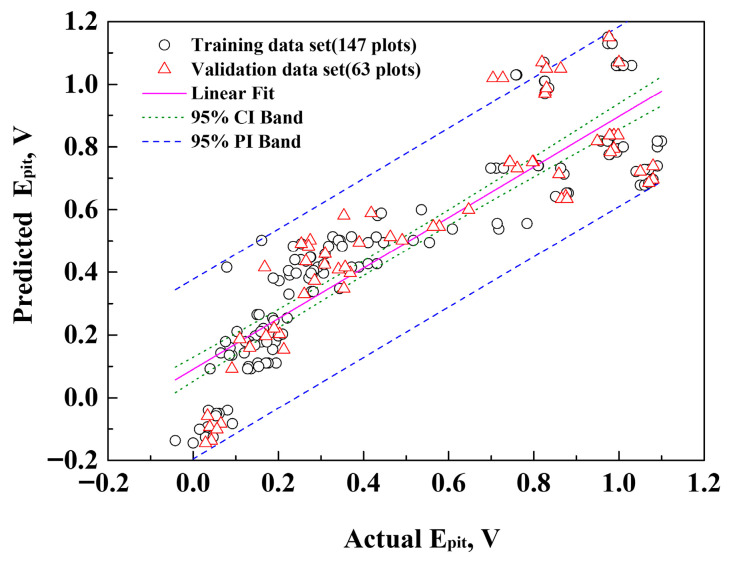
Comparison of actual and predicted values for critical pitting potential: training data set: 147 plots, validation data set: 63 plots.

Figure 5 illustrates the probability plot between the actual and predicted data. In the probability plot, the green dotted line represents the 95% confidence interval, and the blue dashed line represents the 95% prediction interval. The confidence interval serves as a criterion for assessing the uncertainty of the regression function based on statistically limited data or data with many residuals [29]. The prediction interval serves as a criterion for assessing the uncertainty of new data values in a curve affected by noise [30]. In other words, the confidence and prediction intervals represent the probabilistic reliability between the actual and predicted values. This confirms the high reliability and significance of predicting the pitting potential under complex environmental conditions of the factors.

### 3.4. Prediction of the Polarization Curve Using ANN

The linear functions modeled through full factorial design and analysis of variance demonstrated considerable reliability in predicting the pitting potential. However, there are limitations in modeling dynamic behaviors such as nonlinear polarization curves. ANN are highly suitable techniques for modeling data with nonlinear structures. Therefore, polarization curves at each factor level were predicted using an ANN model. Generally, ANN can perform accurate predictions with wide and deep architectures [31,32]. However, computational time and cost increase, and overfitting to the training data may occur. Therefore, selecting the optimal activation function and neuron architecture suitable for the type and complexity of the data is necessary. In previous studies on predicting dynamic polarization curves in complex environments, Hu et al. [30] used an ANN model consisting of a single hidden layer (sigmoid function) with 5 inputs and 50 neurons, and an output layer with 2 neurons. Kamrunnahar et al. [33] used a model with 7 inputs, triple hidden layers (tangent sigmoid function) consisting of 100, 100, and 50 neurons, and an output layer with 4 neurons. Wang et al. [34] employed a model with 5 inputs, double hidden layers (tangent sigmoid function) with 35 and 35 neurons, and an output layer with 4 neurons. As such, since the optimal activation function and number of neurons in hidden layers depend on the type and complexity of the data, it is very difficult to specify them. Therefore, the optimal ANN structure must be selected through numerous trials and errors. To select the optimal activation function and number of nodes suitable for the data type, activation functions such as sigmoid and tanh were used, and the ANN model was validated across various combinations.

$$\mathrm{sigmoid}\;\mathrm{activation}\;\mathrm{function}:\;f\left(x\right)=\frac{1}{1+e^{-x}}=\frac{e^{x}}{e^{x}+1}$$

$$\mathrm{hyperbolic}\;\mathrm{tangent}\;\mathrm{activation}\;\mathrm{function}:\;f\left(x\right)=\frac{e^{x}-e^{-x}}{e^{x}+e^{-x}}$$

**Figure 6:**
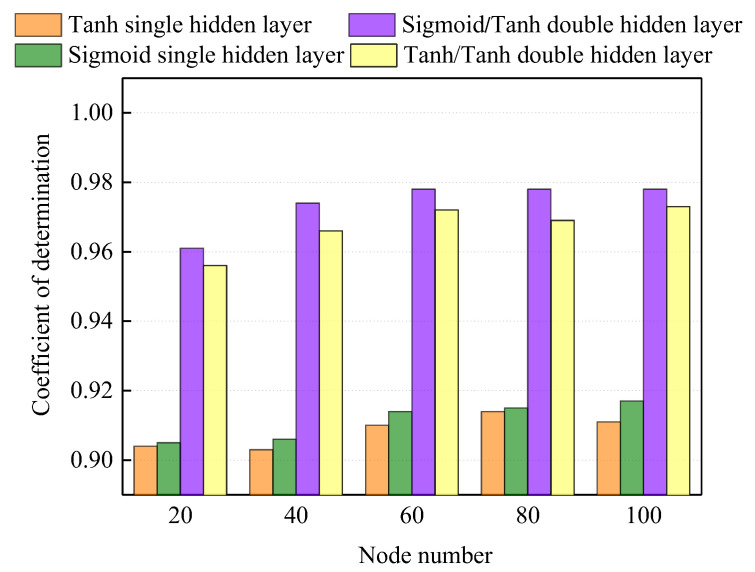
R^2^ of ANN model with different activation functions and node numbers.

Figure 6 shows R^2^ of 0.9 or higher among various ANN structures. The R^2^ tended to increase with the number of neurons. However, it plateaued beyond 60 neurons. Therefore, considering computational cost, an optimal number of 60 neurons was selected. Additionally, the overall structure employing sequential activation functions specifically sigmoid and tanh in a 5-60-60-1 architecture exhibited the highest R^2^ of 0.972.

**Figure 7:**
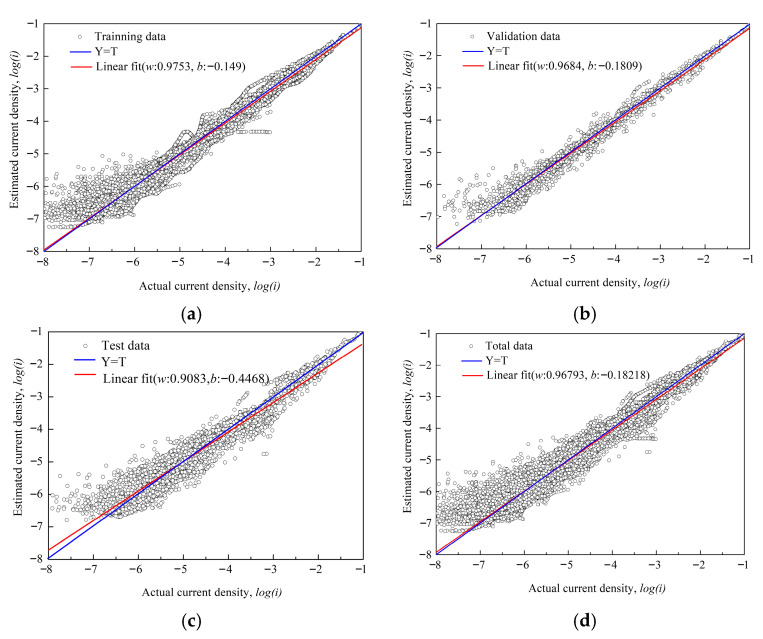
Comparison of actual and ANN predicted values for *log(i)*: (**a**) training data set: 53,964 plots (**b**) validation data set: 5995 plots (**c**) test data set 6661 plots, (**d**) total data set: 66,602 plots.

Figure 7 presents a comparison between actual and predicted values for 53,964 training data points, 5995 validation data points, and 6661 test data points. In the graph, the fit line (blue solid line) represents a perfect 100% agreement between actual and predicted data. Overall, the data points were distributed along the fit line, with coefficients of determination R^2^ of 0.975, 0.968, and 0.908 for the training, validation, and test sets, respectively.

In Figure 7a,b, the points showed relatively high dispersion at low current densities; however, in the current density regions above 1 × 10^−6^ A/cm^2^ where passive regions and pitting behavior occur in each polarization test—the dispersion was relatively close to the fit line.

**Figure 8:**
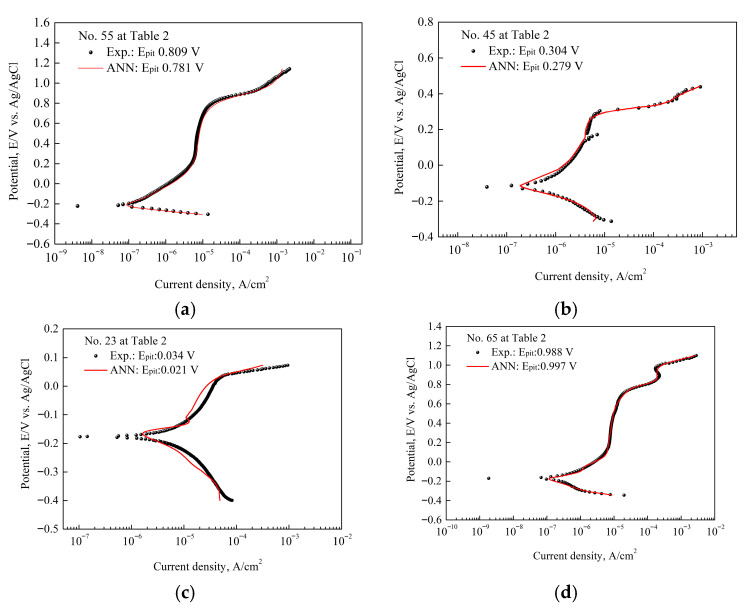
Experimental and ANN predicted polarization curves for some training data sets. These conditions are included in Table 3.

Figure 8 compares the actual and predicted curves under test conditions included in the ANN training process to verify the training, validation, and test data. The ANN exhibited a very high degree of fit to the training data. The experimental and predicted curves were very similar in shape and matched very accurately in characteristic values such as OCP.

**Figure 9:**
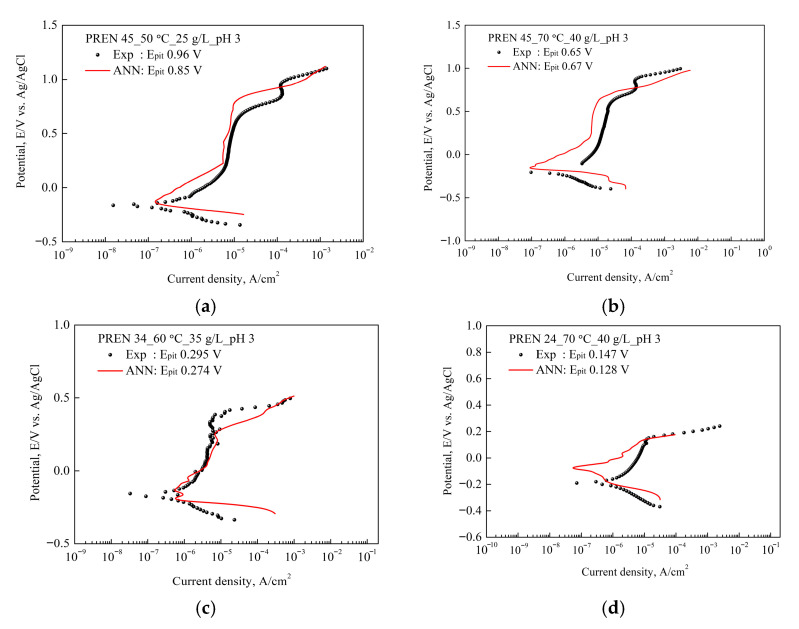
Experimental and ANN predicted polarization curves. These conditions are not included in Table 3 and the pitting potential. These results confirm the predictive performance of the ANN for the dynamic behavior of flexible and complex relationships like electrochemical polarization.

Figure 9 compares the experimental and predicted curves for new test conditions not included in the ANN model training. In Figure 9d, a slight deviation occurred between the two curves; however, the overall behavior and the prediction of the critical pitting potential showed a considerable tendency to match. Considering the inherent flexibility and complexity of electrochemical polarization tests, the predictive performance is judged to be of a fairly high level of reliability. Additionally, excluding the pitting resistance equivalent number (PREN), only 27 environmental variables were provided for each material, which can be considered data for some limited environments. It is anticipated that if the ANN model is trained with data from various levels, it could exhibit very high predictive performance.

In the experimental polarization curves, metastable pitting points were observed in Figure 8b and Figure 9c, and in some experimental data, two distinct pitting stages appeared in Figure 8d and Figure 9a,b. These features are typical of stainless steels due to their multiphase microstructures and varying electrochemical behavior. By contrast, the ANN-predicted curves smooth out such fluctuations and sharp transitions, since the model is data-driven and relies on pattern recognition rather than capturing electrochemical mechanisms. As a result, ANN predictions reproduce overall polarization trends; nevertheless, they do not adequately reflect localized phenomena such as metastable pit initiation, phase-dependent breakdown, or passivity loss. Although oversimplified representation is a limitation of machine learning approaches, it also emphasizes their complementary role in providing reliable general trends of corrosion behavior.

These findings confirm the predictive performance of the ANN for the dynamic behavior of electrochemical polarization under complex conditions. In addition, the applicability of the proposed methodology extends to the pitting corrosion examined in this study. Because both the regression and ANN models are fundamentally data-driven, they can be adapted to analyze other localized corrosion mechanisms, such as crevice corrosion or stress corrosion cracking. This flexibility highlights the potential of the approach as a versatile tool in corrosion prediction. Nevertheless, several limitations must be acknowledged. The present work was performed under controlled laboratory conditions and focused exclusively on pitting corrosion, which restricts direct extrapolation to other corrosion forms or complex field environments. Furthermore, uniform corrosion and microbiologically influenced corrosion may involve additional chemical or biological factors that are not fully represented by polarization data and thus may require further refinement of the model and broader datasets.

## 4. Conclusions

In this study, statistical approaches utilizing linear regression and ANN were applied to predict the pitting potential and polarization curves of austenitic stainless steels (316L, 904L, and AL-6XN) under various environmental variables, including temperature, Cl^−^ ion concentration, and pH.

The ANOVA confirmed that each input variable PREN ranging from 24 to 45, temperature from 30 to 90 °C, Cl^−^ ion concentration from 20 to 40 g/L, and pH from 2 to 6 had a significant effect on the critical pitting potential. The influence order was determined to be PREN, temperature, pH, and Cl^−^ ion concentration.

A linear regression model was established using significant factors and interactions identified at the 95% confidence level from ANOVA. The linear regression model exhibited a considerable predictive performance (R^2^ = 0.789) in forecasting the critical pitting potential.

To predict potentiodynamic polarization curves, an ANN based on supervised learning with backpropagation was employed. The ANN model demonstrated a remarkably high predictive performance (R^2^ = 0.972) for polarization curves in complex corrosion environments. The polarization curves predicted by the ANN model showed significant reliability in estimating electrochemical parameters such as corrosion current, corrosion potential, and pitting potential.

The results provide a valuable tool for predicting and understanding the corrosion behavior of stainless steels and are expected to contribute to decision-making in corrosion prevention and material selection in the future.

## Figures and Tables

**Table 1:** Chemical composition (wt.%) for steels.

Material	PREN	C	Si	Mn	Ni	Cr	Mo	Cu	N	Fe
316L	24	0.019	0.58	1.07	10.23	16.76	2.03	0.3	-	Bal.
904L	34	0.02	0.64	1.53	24	19.27	4.21	1.3	0.04	Bal.
AL-6XN	45	0.016	0.63	0.28	23.8	21.7	6.6	0.19	0.21	Bal.

**Table 2:** Experimental independent factor and their levels.

Factors	Unit	Level (Normalized Value)		
PREN (A)		24 (0)	34 (0.5)	45 (1)
Temperature (B)	°C	30 (0)	60 (0.5)	90 (1)
Cl^−^ concentration(C)	g/L	20 (0)	30 (0.5)	40 (1)
pH (D)		2 (0)	4 (0.5)	6 (1)

**Table 3:** Full factorial design matrix and experiment results.

Exp. No. (PREN)			Factor			E_corr_., V	E_pit_, V	E_range_, V
			Temp., °C	Cl^−^, g/L	pH			
1 (24)	28 (34)	55 (45)	30	20	2	0.072	0.354	0.282
2 (24)	29 (34)	56 (45)	30	20	4	−0.029	0.279	0.308
3 (24)	30 (34)	57 (45)	30	20	6	−0.043	0.265	0.308
4 (24)	31 (34)	58 (45)	30	30	2	−0.085	0.277	0.362
5 (24)	32 (34)	59 (45)	30	30	4	−0.07	0.272	0.342
6 (24)	33 (34)	60 (45)	30	30	6	−0.081	0.309	0.39
7 (24)	34 (34)	61 (45)	30	40	2	−0.098	0.225	0.323
8 (24)	35 (34)	62 (45)	30	40	4	−0.066	0.285	0.351
9 (24)	36 (34)	63 (45)	30	40	6	0.034	0.357	0.323
10 (24)	37 (34)	64 (45)	60	20	2	−0.13	0.152	0.282
11 (24)	38 (34)	65 (45)	60	20	4	−0.062	0.213	0.275
12 (24)	39 (34)	66 (45)	60	20	6	−0.118	0.146	0.264
13 (24)	40 (34)	67 (45)	60	30	2	−0.167	0.13	0.297
14 (24)	41 (34)	68 (45)	60	30	4	−0.116	0.065	0.181
15 (24)	42 (34)	69 (45)	60	30	6	−0.108	0.11	0.218
16 (24)	43 (34)	70 (45)	60	40	2	−0.117	0.091	0.208
17 (24)	44 (34)	71 (45)	60	40	4	−0.114	0.085	0.199
18 (24)	45 (34)	72 (45)	60	40	6	−0.295	0.123	0.418
19 (24)	46 (34)	73 (45)	90	20	2	−0.149	0.029	0.178
20 (24)	47 (34)	74 (45)	90	20	4	−0.157	0.065	0.222
21 (24)	48 (34)	75 (45)	90	20	6	−0.134	0.081	0.215
22 (24)	49 (34)	76 (45)	90	30	2	−0.166	0.043	0.209
23 (24)	50 (34)	77 (45)	90	30	4	−0.149	0.034	0.183
24 (24)	51 (34)	78 (45)	90	30	6	−0.114	0.055	0.169
25 (24)	52 (34)	79 (45)	90	40	2	−0.164	0	0.164
26 (24)	53 (34)	80 (45)	90	40	4	−0.107	0.015	0.122
27 (24)	54 (34)	81 (45)	90	40	6	−0.148	0.034	0.182

**Table 4:** Coefficient of determination according to the interaction term of the multiple linear regression model.

Term	R^2^	Adjusted-R^2^
Linear	0.789	0.777
Quadratic	0.973	0.966
Cubic	0.993	0.989
Quaternary	0.996	0.993

**Table 5:** Result of ANOVA on the full factorial design.

Source	DF	Adj SS	Adj MS	F-Value	*p*-Value
Model	15	19.5002	1.30001	272.54	0.000
Blocks	1	0.0000	0.00004	0.01	0.926
Linear	6	15.8551	2.64252	554.00	0.000
A	2	10.0457	5.02287	1053.03	0.000
B	2	5.6797	2.83987	595.37	0.000
D	2	0.1296	0.06480	13.59	0.000
2-Way Interactions	8	3.6450	0.45563	95.52	0.000
A·B	4	3.5707	0.89267	187.15	0.000
A·D	4	0.0744	0.01859	3.90	0.005
Error	146	0.6964	0.00477		
Total	161	20.1966			
R^2^			Adjusted-R^2^		
96.55%			96.20%		

**Table 6:** Regression models and their determination coefficient for critical pitting potential.

Term	Regression Model		
	R^2^	R^2^-Adjust	R^2^-Prediction
Linear	E_pit_ = 0.381+0.6085A−0.458B−0.031C+0.063D		
	0.779	0.774	0.76
Quadratic	E_pit_ = 0.355 + 0.5890 A − 0.4017 B − 0.064 D − 0.036 A · B + 0.0753 A · B − 0.0767 B · D		
	0.783	0.768	0.756

## Data Availability

The original contributions presented in this study are included in the article. Further inquiries can be directed to the corresponding author.

## Abbreviations

The following abbreviations are used in this manuscript:

ANN	Artificial Neural Network
PREN	Pitting Resistance Equivalent Number
ANOVA	Analysis of Variance
OCP	Open Circuit Potential
